# Application effect of head-mounted mixed reality device combined with 3D printing model in neurosurgery ventricular and hematoma puncture training

**DOI:** 10.1186/s12909-023-04659-6

**Published:** 2023-09-18

**Authors:** Yilong Peng, Zhengyuan Xie, Shaoai Chen, Yi Wu, Jiajun Dong, Jinhong Li, Jinlang He, Xiaolei Chen, Hongzhi Gao

**Affiliations:** 1 Department of Neurosurgery, The Second Affiliated Hospital of Fujian Medical University, Quanzhou, 362000 Fujian China; 2 Department of Neurosurgery, Jiangmen Central Hospital, Jiangmen, 529030 Guangdong China; 3 Department of Dermatology, Jiangmen Central Hospital, Jiangmen, 529030 Guangdong China; 4 Department of Neurosurgery, First Medical Center, Chinese People’s Liberation Army General Hospital, Beijing, 100853 China

**Keywords:** HoloLens, MR technology, 3D printing model, Training system

## Abstract

**Background:**

The purpose of this study was to explore the applicability of application effect of head-mounted mixed reality (MR) equipment combined with a three-dimensional (3D) printed model in neurosurgical ventricular and haematoma puncture training.

**Methods:**

Digital Imaging and Communications in Medicine (DICOM) format image data of two patients with common neurosurgical diseases (hydrocephalus and basal ganglia haemorrhage) were imported into 3D Slicer software for 3D reconstruction, saved, and printed using 3D printing to produce a 1:1-sized head model with real person characteristics. The required model (brain ventricle, haematoma, puncture path, etc.) was constructed and imported into the head-mounted MR device, HoloLens, and a risk-free, visual, and repeatable system was designed for the training of junior physicians. A total of 16 junior physicians who studied under this specialty from September 2020 to March 2022 were selected as the research participants, and the applicability of the equipment and model during training was evaluated with assessment score sheets and questionnaires after training.

**Results:**

According to results of the assessment and questionnaire, the doctors trained by this system are more familiar with the localization of the lateral anterior ventricle horn puncture and the common endoscopic surgery for basal ganglia haemorrhage, as well as more confident in the mastery of these two operations than the traditional training methods.

**Conclusions:**

The use of head-mounted MR equipment combined with 3D printing models can provide an ideal platform for the operation training of young doctors. Through holographic images created from the combination of virtual and real images, operators can be better immersed in the operation process and deepen their understanding of the operation and related anatomical structures. The 3D printed model can be repeatedly reproduced so that doctors can master the technology, learn from mistakes, better achieve the purpose of teaching and training, and improve the effect of training.

## Introduction

Neurosurgery has a long learning curve, and because mastery of its surgical skill often require years of training, thereby reducing the risks associated with neurosurgical treatment. At present, most of the trainings in China are held through theoretical teaching, reading anatomical maps, assessing human specimens, and observing operations performed by superior physicians. Finally, the skills are gradually accumulated and mastered under the guidance of superior physicians. Recently, with the increasing application of digital image processing technology in the medical field, technologies such as virtual reality (VR), augmented reality (AR), and three-dimensional (3D) printing have been gradually introduced into modern medical education and training. As a result, there have been changes in teaching neurosurgical residents [1–6]. VR is a purely digital picture, and AR is simply an overlay of virtual pictures in the real environment. Mixed reality technology (MR) is an advanced technology that was developed on the basis of VR and AR technology. It can superimpose virtual images on real world images and build an interactive feedback information loop between the virtual world, real world and user to enhance the sense of reality of the user experience [7, 8]. This technology has been gradually applied in the medical field. In clinical surgery, it can not only promote the accurate implementation of the real surgical process but can also facilitate the learning and practice of surgical skills in a virtual surgical environment, which has broad application prospects. 3D printed models are constructed layer-by-layer using computers and can be used to produce individual surgical training models with anatomical details [9]. Neurosurgeons must often undertake surgical positioning for the basal ganglia haemorrhage and Ventricle puncture [10]; young physicians must perform precise puncturing of the anterior horn of the lateral ventricle before being allowed on duty independently. However, the process of accurate puncturing is challenging for beginners. The consequence of puncture failure can be disastrous. Unfortunately, traditional teaching approaches have limitations in terms of its training efficacy and ability to promote self-confidence in new neurosurgeons. A new and effective teaching method is currently needed. This study describes the training system that was designed on the basis of the use of a head-mounted MR device with a 3D printed model and discusses their applicability in the training of puncture operations of lateral ventricles and the basal ganglia for treatment of haematoma in neurosurgery.

## Materials and methods

### Participant characteristics

A total of 16 junior physicians who studied in the undergraduate room from September 2020 to March 2022 were selected as the research participants and were randomly divided into two groups with eight people in each group. Group A was the control group, and Group B was the research group. Before training, all physicians acquired relevant knowledge from related textbooks and observed the work of superior physicians. None of them had any experience in independently performing lateral ventricle puncture and basal ganglia haematoma puncture. This study was approved by the Ethics Committee of the Jiangmen Central Hospital (approval no. [2021] 17), and all the participants provided written informed consent.

### Design of the training system

The original 3D reconstruction and model printing data used to reconstruct the cranial image were obtained through the picture archiving and communication software system of the hospital network. Head computed tomography (CT) was performed (Siemens SOMATOM Force dual 96-slice, Germany). The scanning parameters were as follows: tube voltage, 90 kVp; window width, 300; window level, 40; matrix, 512 × 512; field of view, 23 cm; slice thickness, 0.675 mm; the export format was medical numbers, Digital Imaging and Communications in Medicine (DICOM). The images from the two patients were reconstructed and uploaded into 3D Slicer software (http://www.slicer.org/, free opensource software) by the same deputy chief physician. The procedure was as follows:

3D Slicer software (3D Slicer 4.10.1) was run on the computer, and the CT data were imported into the software in DICOM format. Through the relevant programs in the Segment Editor module, two models with a size of 1:1 was constructed based on real person characteristics, including the skull, skin, and associated anatomical structures.

**Fig. 1:**
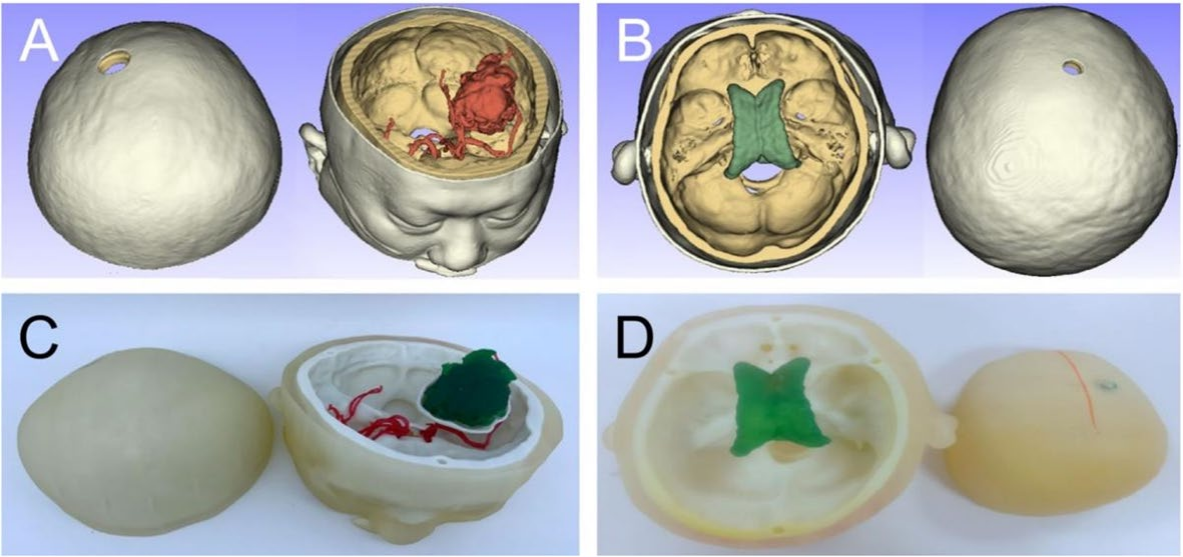
Real person 1:1-sized 3D printing model. **A**, **B** Model of basal ganglia intracerebral hemorrhage and hydrocephalus reconstructed using 3D Slicer, with skin in white, skull in yellow, blood vessels and hematoma in red, and ventricle in green. **C**, **D** The model printed using 1:1 3D reconstruction; dark green is hematoma, red is blood vessel, and light green is ventricle

The head cover and skull base are separated, which can be used to observe the condition inside. In the hydrocephalus model, the ventricle must be reconstructed, and a bone hole should be made. In the cerebral haemorrhage model, support for the haematoma and fenestration of the ipsilateral skull were included, which were sealed with plasticine before the operation. Finally, the file was saved in 3D printing format (.stl), and the corresponding head cover and skull base were reconstructed using photosensitive resin material (hard + soft) via a 3D printing machine (J401pro of Zhuhai Senna Printing Technology Co., Ltd., Zhuhai City of Guangdong province, China). The different parts of the 3D printed model were attached using magnets (Fig. 1).

### Import and operation of the hybrid display device model

**Fig. 2:**
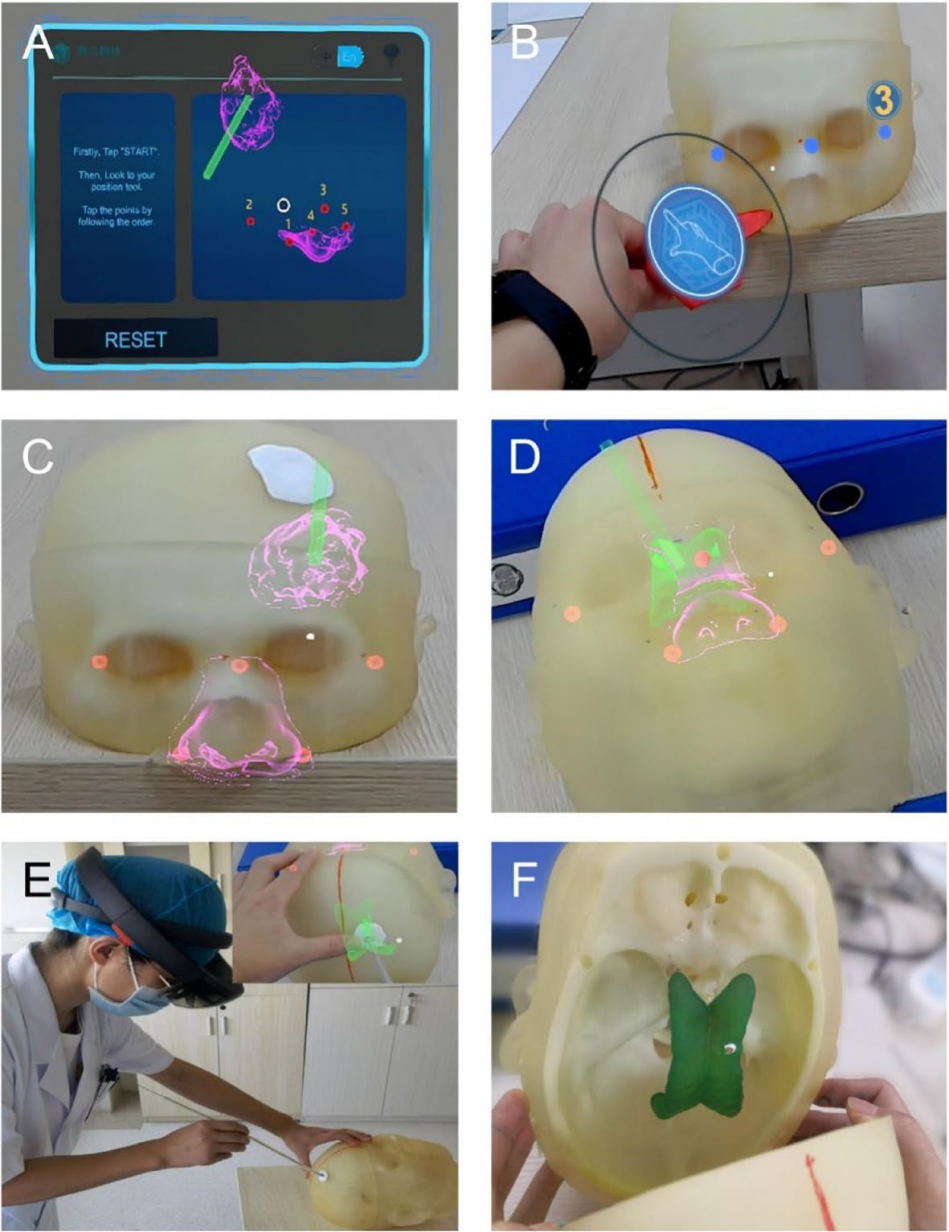
Import of hybrid display device model and operation training with 3D printing model. **A** Download the reconstructed 3D model to the HoloLens device by installing the HoloLens application MRN. **B** Register the 3D printed model so that the reconstructed model image is superimposed on the head model. **C** A successfully registered hematoma model can show the green puncture path in the center of the red hematoma. **D** Successfully registered hydrocephalus model; the green one is the ventricle and puncture path. **E** Operation training for young doctors to perform ventricular puncture under the guidance of holographic images of mixed reality equipment. **F** The position of the white paint at the tip of the drainage tube on the green ventricle during puncture

Upload the 3D reconstructed model of the ventricle, haematoma, puncture path, and anatomical structure to ensure that it matches the head model to the cloud server through Unity (version 3.1). After the upload is successful, open and install the HoloLens application mixed-reality neuronavigation to download the model to the HoloLens (Microsoft Corp.) device (the application was independently developed by the team of Professor Chen Xiaolei of the Chinese People's Liberation Army General Hospital). The visual model hologram displayed by a small computer screen placed in front of the eye can be clearly seen through the high-definition lens. The 3D printed model is registered by an instructor who is familiar with the operation of the HoloLens so that the reconstructed model image is superimposed on the head model and subsequently checked to determine whether the registration matches the relative anatomical position of the head. This step is equivalent to the registration of navigation; the head model cannot be moved after satisfactory positioning. After successful registration, the reconstructed ventricle, haematoma, and designed puncture path can be seen on the head model (Fig. 2).

### Puncture operation training

Group A was taught by senior physicians with demonstrations on skull specimens using traditional methods to study the puncture of the anterior horn of the lateral ventricle and conventional approaches for endoscopic surgery for basal ganglia haemorrhage. Puncture of the basal ganglia is the first step of endoscopic surgery for haemorrhage. The puncture points of the anterior horn of the lateral ventricle and basal ganglia are the same—Kocher’s point [11], which is located 1–2 cm anterior to the coronal suture and 2–3 cm lateral to the midline (approximately at the mid-pupillary line) and approximately 11 cm posterior to the nasion, or 10 cm posterior from the glabella. However, the puncture direction of these two operations is different. The puncture direction of the anterior horn of the lateral ventricle is toward the middle point of the bilateral external auditory canal connection line, while the puncture direction of basal ganglia hemorrhage is toward the long axis of the hematoma. The anterior horn of the ventricle can be punctured from two directions. The midpoint of the line connecting the lateral external auditory canal and the midline of the basal ganglia intracerebral haemorrhage was parallel to the midline, and the long axis of the haematoma was punctured as far as possible. Operation training is subsequently performed on the 3D printed model. The training time is 2 weeks, including one theoretical lecture, two operational exercises, and finally, an assessment. Group B was trained using the same teaching approach as Group A. However, in Group B, head-mounted MR equipment—Microsoft HoloLens— was added and used in combination with the 3D printed models for operation training and subsequent assessments. The training time and number of operation exercises were the same as those of Group A. Assessment criteria were as follows: the colour paint on the puncture tube was subjected to contact with the lesion model; the ventricle puncture was at the frontal angle, and the haematoma was at the middle. (Fig. 2).

### Assessment score and satisfaction questionnaire

There are 5 individual items in the assessment, each with a full score of 5 points; more than 20 points is excellent, 15–20 is good, and < 15 is unqualified. The items included:

① Placement of the body
② Positioning on the model
③ Depth of the puncture
④ Accuracy of the puncture position
⑤ Overall understanding of the operation.

The satisfaction questionnaire had the following 5 items:

① Training method
② Training content
③ Training effect
④ Training process experience
⑤ Self-confidence improvement after training.

The grading standard was the same as the assessment grading.

### Statistical analysis

Statistical Package for the Social Sciences (SPSS; version 21.0; SPSS Inc., Chicago, IL, USA) software was used for statistical analysis, and measurement data are expressed as the mean ± standard error (x ± s). Mann–Whitney U tests, rank-sum tests, and two independent samples t tests were used for the overall understanding of the results of the questionnaire. *P* < 0.05 was considered statistically significant.

## Results

**Fig. 3:**
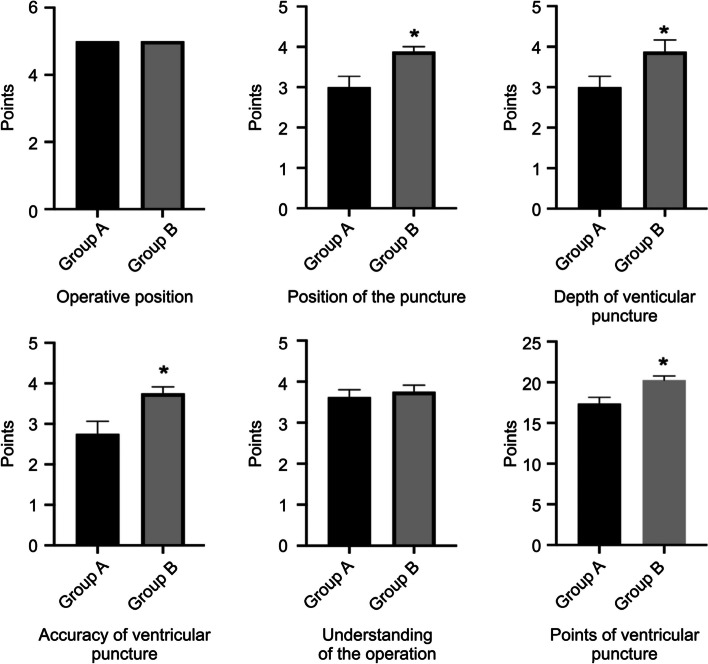
The assessment scores of the two groups of physicians in the ventricular puncture operation

**Fig. 4:**
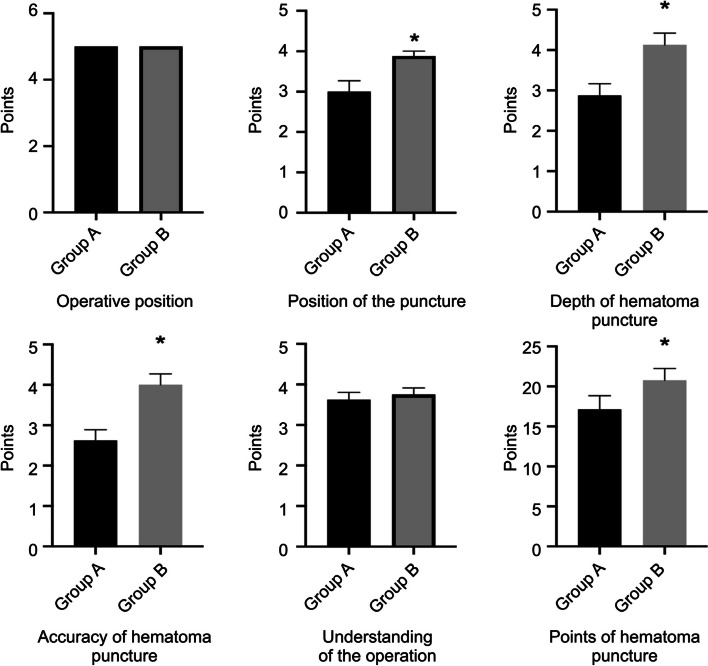
The assessment scores of the two groups of physicians in the hematoma puncture operation

After the operation training with the head-mounted MR device and the 3D printed model, the doctors of both groups underwent assessment. For positioning on the model, Group B scored 3.875 ± 0.125, which was higher than that of Group A (3 ± 0.267) (*P* < 0.05). The score for puncture depth during ventricle puncture in Group B was 3.875 ± 0.295, which was higher than that in Group A (3 ± 0.267) (*P* < 0.05). The score for puncture depth during basal ganglia haematoma puncture in Group B was 4.125 ± 0.295, which was higher than that in Group A, 2.875 ± 0.295 (*P* < 0.05). Regarding the score for puncture accuracy in Group B was 3.75 ± 0.164, which was higher than that in Group A, 2.75 ± 0.313 (*P* < 0.05), and the score for haematoma puncture in Group B was 4 ± 0.267, which was higher than that in Group A, 2.625 ± 0.263 (*P* < 0.05). The scores of Group B in terms of the positioning on the model, depth of puncture, and position accuracy were higher than those of Group A, and the differences were statistically significant (*P* < 0.05). There was no significant difference between Groups A and B in terms of body position placement and overall understanding of the operation (*P* > 0.05). The final score of the operation assessment in Group B was 20.25 ± 0.526 in terms of puncturing the ventricle, which was significantly different from the score in Group A, which was 17.38 ± 0.778 (*P* < 0.01). The score of haematoma puncture in Group B was 20.75 ± 0.526, which was significantly higher than that in Group A, which was 17.13 ± 0.611 (*P* < 0.01) (Figs. 3 and 4).

**Fig. 5:**
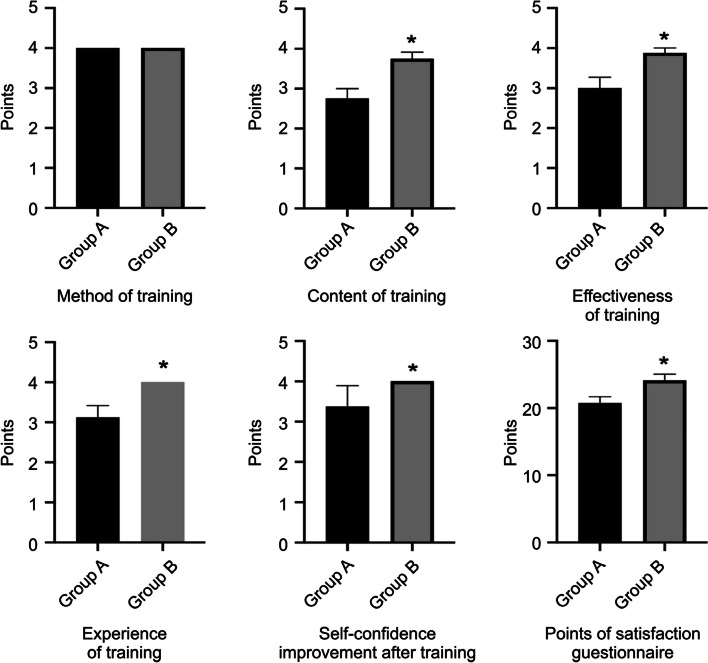
The scores of the two groups of physicians in the questionnaire

In the questionnaire survey, the scores of Group B in terms of training content, 3.75 ± 0.164; training effect, 3.75 ± 0.164; experience in the training process, 4 ± 0; and the improvement of self-confidence from training, 4 ± 0 were significantly higher than those of Group A, whose scores were training content, 2.75 ± 0.25; training effect, 3 ± 0.267; experience in the training process, 3.125 ± 0.295; and self-confidence improvement from training, 3.375 ± 0.183 (*P* < 0.05). There was no significant difference between Groups A and B in terms of training methods (*P* > 0.05). The total score of the questionnaire in Group B was 24.13 ± 0.934, which was significantly higher than that of Group A (20.75 ± 0.05) (*P* < 0.05) (Fig. 5).

## Discussion

The puncture of the anterior horn of the lateral ventricle and the surgical treatment of basal ganglia intracerebral haemorrhage are the two surgical operations frequently encountered by neurosurgeons on duty and require mastery of their basic skills. Compared with other operations in neurosurgery, puncturing the anterior horn of the lateral ventricle is relatively simple; however, it is not easy to puncture in the correct place, thus suggesting a certain learning curve. As the basal ganglia is a common site of hypertensive intracerebral haemorrhage, hypertensive intracerebral haemorrhage in the basal ganglia is one of the most severe diseases that requires neurosurgical intervention. Recently, with the popularization and application of neuroendoscopy in clinical practice, endoscopic haematoma evacuation has been gradually performed in China and has become one of the surgical methods for hypertensive intracerebral haemorrhage. Therefore, in this study, anterior horn puncture of the lateral ventricle and a conventional endoscopic surgical approach for basal ganglia haemorrhage were chosen to treat to train young doctors, with the aim of improving their surgical skills, shortening the learning curve, reducing the risk of surgery, and improving the success rate of surgery.

**Fig. 6:**
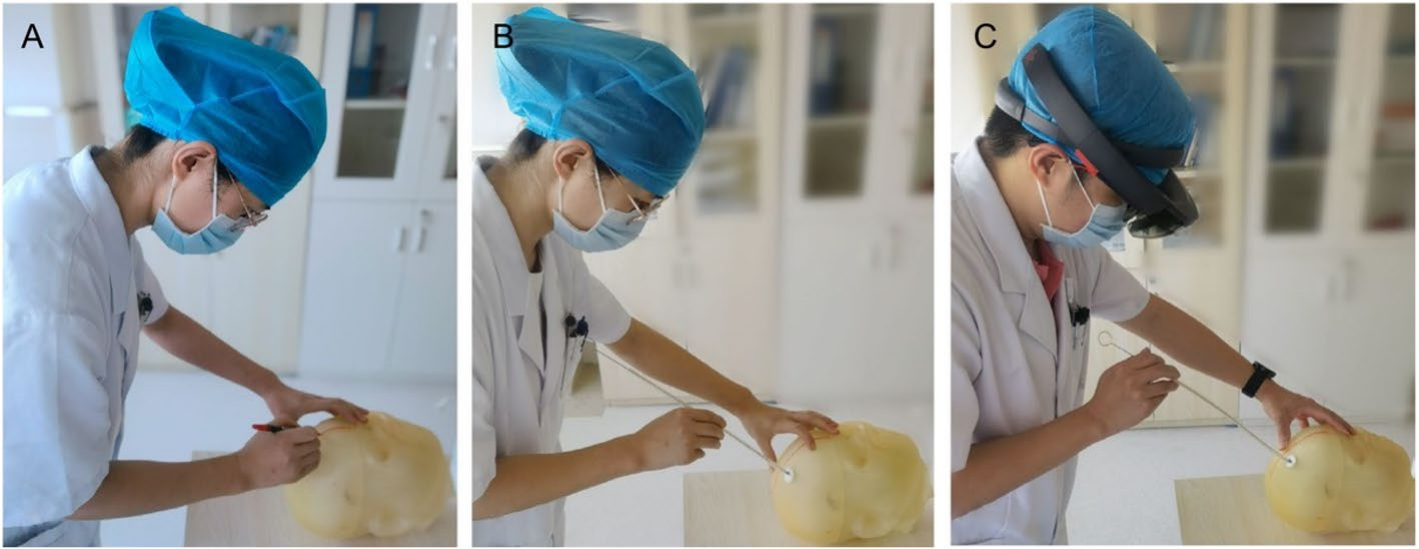
Line drawing, positioning, puncturing, and other operations on the printed model. **A** A young doctor draws lines and positions on the model. **B**, **C**) Physicians with different seniority conduct puncture operation training on the model

Currently, in addition to traditional theoretical teaching, domestic neurosurgical training provides trainees to practice performing the operation by participating in relevant training courses. For most young doctors, neurosurgical skills are typically obtained by observing the operation performed by superior doctors and gradually accumulating experience under their guidance, but this may increase the occurrence of complications and cause ethical issues [12–15]. 3D printing technology appeared in the 1990s and has been widely used in the medical, military, and aerospace industries and other fields [16, 17]. Its emergence makes personalized surgical training possible. In this study, a 3D 1:1 head model is printed using real patient data, including scalp, skull, and relevant anatomical structures because such a model almost certainly shows the real situation of the patient's head. The operator can draw lines and plan the position of the puncture on the model, thereby simulating real surgical operations, without any risks. This method is repeatable and can evaluate the effect of the operation more realistically (Fig. 6).

Although the printing price of the head model is relatively expensive at present, training based on a head-mounted MR device and a 3D printed model is still a good choice. Considering the difficulty in obtaining the cadaver head, evaluating the effect of the operation and the imprecision of the simulation model. Through relevant data analysis, it was found that the scores of Group B were higher than those of Group A in terms of positioning on the model, depth of puncture, and position accuracy. In terms of the total score, the doctors in Group A had a general grasp of the two operations after training. The situation was not bad; the total score was 17.38 ± 0.778 points but compared with the total score of Group B of 20.25 ± 0.526 points, there was a significant difference (Table 1). The analysis revealed that the lower score was mainly observed in the accuracy of the puncture location. Physicians in Group A had little problem in selecting the positioning point, but there were still cases where the puncture was unsuccessful or the location was not ideal during the actual operation.

**Table 1:** Groups A and B operation assessment score comparison table

			**Assessment Score**					
			Placement of the body position	Positioning of the operation on the model	Depth of the puncture	Accuracy of the puncture position	Overall understanding of the operation	Final score
**score**	Puncture of ventricle	Group A	5 ± 0	3 ± 0.267	3 ± 0.267	2.75 ± 0.313	3.625 ± 0.183	17.38 ± 0.778
		Group B	5 ± 0	3.875 ± 0.125	3.875 ± 0.295	3.75 ± 0.164	3.75 ± 0.164	20.25 ± 0.526
	*P*		> 0.05	< 0.05	< 0.05	< 0.05	> 0.05	< 0.05
	Puncture of hematoma	Group A	5 ± 0	3 ± 0.267	2.875 ± 0.295	2.625 ± 0.263	3.625 ± 0.183	17.13 ± 0.611
		Group B	5 ± 0	3.875 ± 0.125	4.125 ± 0.295	4 ± 0.267	3.75 ± 0.164	20.75 ± 0.526
	*P*		> 0.05	< 0.05	< 0.05	< 0.05	> 0.05	< 0.05

Data are expressed as mean ± standard error (x ± s)

A study analysed the reasons and reported that young doctors are not familiar with the relevant anatomy, have poor understanding of and no experience in spatial positioning for puncture during the operation, are not proficient enough, and lack self-confidence [18]. Therefore, in the follow-up questionnaire survey, it was found that young doctors were quite satisfied with the content and effect of this kind of operation training and believed that after training, they could master the whole operation process and thereby improve their self-confidence.

With the development of medical imaging technology and equipment, the application of VR and AR has improved surgical planning [19, 20]. Since 2016, the author has been utilizing VR and AR technologies to develop preoperative plans and implement them during surgeries. These techniques also play an important role in medical education [21]. VR and AR in MR is a further development [7]. It uses holographic projection technology to superimpose the hologram into the user's field of vision so that the operator can maintain the connection between the virtual and real world and interact with the hologram according to their own needs [22, 23]. Microsoft HoloLens is a head-mounted MR device [24] that features a transparent holographic lens, automatic pupil distance calibration, spatial sound, gaze tracking, gesture input, and voice support, running on 2 GB RAM and 64 GB internal storage on Windows 10. With this state-of-the-art technology, we can see a high-definition 3D holographic image of the inside of the patient's head from all angles to carry out relevant positioning and surgical operations. This kind of real-time visual operation training can improve the trainees confidence, help trainees gain operation experience, and lay a solid foundation for future practical operations [25]. On searching the literature for recent reports, it was found that some scholars have begun to use HoloLens to perform ventricular puncture, tumour localization, etc. [26–28] Some scholars apply MR to medical education. Rochlen et al. [29] used MR for peripheral intravenous catheter placement training. Schoeb et al. [30] designed a randomized single-blinded prospective trial using MR to teach ureter catheterization, while others are working on further developing and exploring relevant equipment and software [31–33]. Under the assistance of Professor Chen Xiaolei from the Department of Neurosurgery of the First Medical Center of the Chinese People’s Liberation Army General Hospital, the author of this manuscript improved the positioning method with the help of the registration software developed by his team [34]. The MR device, HoloLens, and 3D printed models were used to design an interactive operation training platform, hoping to provide a more perfect training scheme for the two operations. Judging from the posttraining assessment statistics and questionnaire survey results, compared with the physicians in Group A who simply used the head model, the physicians in Group B who were trained with MR equipment were significantly more accurate in positioning the puncture and required fewer puncture attempts. In terms of training content, training effect, training process experience, and self-confidence improvement, the scores of Group B were higher than those of Group A, and the final total score of Group B was also higher than that of Group A; the difference was statistically significant (Table 2). During the questionnaire survey, the physicians in Group B said that the they felt that training under this new immersive teaching mode provided them with a better “perspective”. Different from traditional training methods, which lack a 3D component and are relatively boring, this training mode enables trainees to experience the operation process in a realistic environment, and they can observe related anatomical structures more intuitively and from multiple angles. In the display of the skull model, selecting the optimal surgical path for repeated operations improves the surgical experience and the understanding of space so that learners can become proficient in using the operation technology, improve their training efficiency, and master the learning curve. In the future, physicians will be more confident and operate more accurately.

**Table 2:** Satisfaction evaluation table of the two groups A and B

		**The satisfaction questionnaire**					
		Training method	Training content	Training effect	Training process experience	Self-confidence improvement after training	Final score
**score**	Group A	4 ± 0	2,75 ± 0.25	3 ± 0.267	3.125 ± 0.295	3.375 ± 0.183	20.75 ± 0.946
	Group B	4 ± 0	3.75 ± 0.164	3.875 ± 0.125	4 ± 0	4 ± 0	24.13 ± 0.934
***P***		> 0.05	< 0.05	< 0.05	< 0.05	< 0.05	< 0.05

Data are expressed as mean ± standard error (x ± s)

Although MR and 3D printing technology have been increasingly used in clinical practice, there are still some limitations, such as the long duration of 3D printing, high printing cost, registration inaccuracy in MR technology, and drift possibility. In addition, operators must work to adapt to the new equipment. Moreover, only 16 junior physicians who studied under this specialty were recruited for this study, which was conducted in a single institution. Given the potential impact of doctors' specialty, age, level, and sex on the results, further institutional participation and a larger sample size are necessary to validate the feasibility and effectiveness of this training approach. However, with advancements in science, equipment updates, and the continuous updating and development of software, the clinical application of these two technologies has broad prospects, especially MR technology, not only in medical education but also in the formulation of surgical plans. It also has unique advantages in patient communication and remote consultation.

## Conclusions

MR technology combined with 3D printing models is a novel and efficient approach that can be applied during training for neurosurgical procedures, including puncturing deep seated haematomas and ventricles, to enhance self-confidence and improve teaching efficiency and effects for young physicians.

## Data Availability

The datasets generated and analysed during the current study are not publicly available due to protection of patients’ privacy, but are available from the corresponding author on reasonable request.
